# Supplementary material for: Are carbon emissions trading and green financial instruments synergistic? -Comprehensive quantitative research based on content analysis
Source: PLoS One. 2024 Mar 7;19(3):e0298601. doi: 10.1371/journal.pone.0298601 (PMC10919601; doi:10.1371/journal.pone.0298601)
Supplement: S1 Data — (ZIP) [file pone.0298601.s001.zip › Data/地级市Z_F工作报告.zip!/╔╜╢1⁄2/╡┬╓▌╩╨/└┤╫╘╣1⁄2╓┌║┼í╛┬φ┐╦╩2╛▌═°í┐_2018.docx]

各位代表：

现在，我代表市人民政府向大会报告工作，请予审议，并请市政协委员和列席会议的同志提出意见。

一、2017年主要工作情况

刚刚过去的一年，在中共德州市委的坚强领导下，在市人大、市政协及社会各界的监督支持下，我们认真贯彻党的十八大、十八届历次全会和党的十九大精神，深入学习贯彻习近平新时代中国特色社会主义思想，全面落实省第十一次党代会和市第十五次党代会部署，紧紧依靠全市人民，从严从实，奋发有为，扎实推进协同发展示范区建设，圆满完成年初确定的主要任务。

（一）始终坚持党的领导，落实上级部署坚定有力

一年来，我们旗帜鲜明讲政治，牢固树立“四个意识”，在思想上政治上行动上同以习近平同志为核心的党中央保持高度一致，认真落实党中央、国务院和省委、省政府及市委的各项工作要求，不搞变通、不打折扣，上级各项部署均在我市得到全面落实。一年来，我们聚精会神抓发展，全面践行新发展理念，经济运行稳中向好，质量效益不断提升。预计实现地区生产总值3200亿元，增长7.5%；一般公共预算收入187.47亿元，增长6.9%，税收占比71.1%，同比提高0.8个百分点；完成固定资产投资2740亿元，增长8%；社会消费品零售总额1539亿元，增长10.3%。三次产业比例由10:48.3:41.7调整为9.7:47.3:43，产业结构持续优化。一年来，我们千方百计守底线，全市各级干部攻坚克难，勇于担当，安全生产、环境保护、脱贫攻坚、金融安全、社会稳定等领域工作均实现了平稳发展，没有出现系统性风险，有力地营造了稳定的内外部环境。

（二）经济实现平稳增长，质量效益明显提升

发展活力不断增强。年初确定的110个市级重点项目顺利实施，完成投资539亿元。预计全年规模以上工业增加值增长7%，工业产品产销率稳定在98%以上，增值税、企业所得税分别增长20%和31%。奇瑞新能源汽车、联孚新能源客车投产下线，威讯半导体等一批重点项目建成投产，丽驰新能源汽车获得工信部专用汽车整车生产资质。企业上市实现历史性突破，金麒麟、金能科技、索通发展和双一科技4家企业在沪深交易所上市，巨丰牧业、德建建科、一飞药业3家企业登陆新三板，6家企业在四板挂牌，实现直接融资304.95亿元。“三航计划”全面实施，全年为“三航”企业办理续贷周转业务10.52亿元，担保融资9.16亿元，收购处置不良资产13.32亿元，新增信贷投放43.8亿元，政府的“黄金信用”为优质企业增信，良好的产业生态正在形成。

动能转换开局良好。落后产能加快去除，我市的产业结构、能源结构持续优化，化工企业转型升级提速，7953家“散乱污”企业被整顿、关停，六大高耗能行业增速全线回落，低于全市平均5个百分点，万元GDP能耗下降8%，超额完成省定任务。工业技改投资同比增长15.4%，增幅居全省第3位。六大战略性新兴产业加速成长，全年实现利润增长13.1%，拉动规模以上工业利润增长2.3个百分点。高新技术产业产值3350亿元，增长18.3%，占规模以上工业产值30.8%，比年初增长1.36个百分点。全年新认定省级高新技术企业50家，新增省级以上科技创新平台48家，德州高新区中央创新区启动建设，泰山集团获批国家级工业设计中心。全国创新创业大赛（新能源及节能环保行业）总决赛落户我市，科技创新对动能转换的促进作用日益显现。人才工作再结硕果，德州鲁北人才改革试验区获省批复，第二届“智汇德州”人才创新创业周成功举办，全年引进省级以上高层次人才团队272个，引进重点高校毕业生数量是上年的2.5倍。2013年以来全市新引进高层次人才数量是历史存量的近10倍。

农业转型发展提速。积极推进“放心农场”建设。粮食喜获丰收，全年粮食总产178亿斤。种植结构进一步优化，调减籽粒玉米21万亩，新增青贮玉米10.8万亩、设施蔬菜5.3万亩。畜牧业加快向集约、规模、生态方向转型，新增标准化养殖场区230个。现代要素加快导入，农业设施装备、科技支撑水平显著增强，全国单体面积最大、技术领先的临邑“百亩级智慧农业大棚”投入运营，14个新棚已启动建设。新型农业经营主体蓬勃发展，全市规模以上农产品加工企业达到840家，主营收入增长8.3%。新产业、新业态发展加速，建成水发田园综合体、德百旅游小镇等各类农业园区263个，培育休闲农业经营主体715个，实现营业收入25亿元。农业绿色发展方式推进加快，农业品牌建设和省级农产品质量安全市创建取得积极进展。全市首批23家“放心农场”正式挂牌，“三品一标”认证产品362个，认证面积达735.5万亩。各县（市、区）均建成农产品质量安全追溯平台，农产品安全合格率稳定在96%以上。

（三）深入实施协同发展战略，开放格局进一步打开

加快区域经济一体化。全面融入京津冀协同发展，与京津重点企业和科研单位的联系更加紧密，成功举办京津冀鲁资本技术交易大会、对接京津冀优质农产品博览会等活动，全年新开工京津冀过亿元项目91个，中国航天科技集团“一库三基地”、中粮产业园、鲁京体育设备生产基地、德州国际会展中心等一批优质项目落地，华润、光大、三一等央企强企进驻德州。基础设施互联互通水平进一步提升，石济客专德州段主线工程开通运营，德州火车站站房改造工程竣工并投入使用，济青高铁齐河段联络线全部完工，省道105济聊线德州段改建工程正式通车，齐河黄河大桥具备通车条件，德州机场进行初步选址。与省会济南、长三角、珠三角地区的经济合作日益紧密。

对外开放呈现新局面。外向型经济逆势上扬，全年完成外贸进出口240.4亿元，增长13.8%，新增备案外商投资企业16家，实际到账外资8.7亿元，增长8.8%。融入“一带一路”取得积极成效，与沿线国家有进出口实绩企业达到530家；成功组织“德州百企下南洋”活动，推动我市企业对外开展产业合作，取得丰硕成果，全年对东盟国家出口增长27%，与益海嘉里、正大集团、发林集团等东盟跨国公司的合作进一步加强。优势企业走出去实现新突破，境外投资6.7亿元，恒源石化马来西亚炼油公司业绩大幅攀升，部分企业拓展海外业务取得积极进展。外经贸新兴业态发展较快，国际服务贸易全面增长，跨境电商业务实现零突破，非洲海外仓顺利运营。中德现代农业创新中心、中荷农业技术卓越中心、巴斯夫现代农业示范基地启动运营。

（四）全面深化改革纵深推进，各项事业蓬勃发展

深入实施国家级、省级改革试点59项，研究出台改革方案70项，改革事业全面发力，多点突破。持续推进“放管服”改革，取消、下放、调整权力事项91项，“三公”经费较去年压减12.9%。在14个部门开展“零跑腿”和“只跑一次”改革试点。市区国地联合办税服务厅建成启用。全面推行综合行政执法体制改革，推进城管、文化、市场监管等领域综合执法。公共资源交易市县一体化平台全面建成，省市县实现互联互通。深化商事制度改革，市场活力不断增强，全年新增市场主体6.2万户，增长14.8%。投融资体制改革走向深入，整合市级经营性资产，新组建市城投集团、交投集团，国有资本布局进一步优化。稳步推进事业单位改革，校长职级制、教师“县管校聘”工作顺利实施，全市40家公立医院全部实施综合改革。扎实推进新型城镇化试点，完成12.1万农业转移人口市民化工作任务，常住人口和户籍人口城镇化率分别达到55.5%、41.99%。深化农业农村改革，农村党支部领办创办土地股份合作社顺利推进，农村土地承包经营权确权登记颁证基本完成，农村产权制度改革试点扎实推进，全市农村土地流转面积330.3万亩，占家庭承包耕地的40%，有力地激发了农业农村发展活力。

（五）民生得到不断改善，社会保持和谐稳定

坚持民生为本，全年民生领域投入286.1亿元，占一般公共预算支出的79.4%。预计城镇和农村居民人均可支配收入分别达到24695元、13338元，增长8.5%和8.9%。坚持精准扶贫、精准脱贫，扶贫质量进一步改善，实施产业扶贫项目234个，全年脱贫人口3.5万人，276个重点村全部摘帽，贫困发生率由2016年初的2.8%下降至0.1%。坚持优先发展教育事业，饱和式投入129亿元对农村中小学“全面改薄”、解决城镇中小学“大班额”问题，投资总量超过前20年的总和，新交付使用学校193所，新招聘教师3681名，如今“最好的建筑是学校，最美的环境在校园”，教育面貌焕然一新。今年所有县（市、区）全部通过国家义务教育基本均衡验收，标志着我市基础教育薄弱的局面得到根本扭转！新增城镇就业连续7年超过6万人，完成就业培训2.1万人，退役士兵安置和权益保障政策全部落实。侯卫东官场笔记社会保障能力进一步提高，职工大病保险启动运行，跨省异地就医联网直接结算全面实现，企业退休人员养老金实现“十三连涨”。加快推进健康德州建设，顺应群众期待，千方百计增加优质医疗服务供给。市人民医院门诊楼、市妇女儿童医院及市中医院病房楼建设快速推进，东部医疗中心完成规划选址。编制完成《德州市养老设施建设和养老产业发展规划》，新增养老床位4026张。对中心城区78个老旧小区实施了改造，群众居住环境得到改善。大力度推进保障性住房建设，全年改造棚户区46495套，是2016年的2.8倍；完成农村危房改造3378户，2444户建档立卡贫困户搬入新居。实施无害化卫生厕所改造26.4万户，累计41万户群众受益。积极改善城乡居民出行条件，中心城区新增新能源公交车300台；新建改造农村公路1520公里，治理隐患路段2032公里，全市县乡公路安全生命防护工程顺利通过省级验收。大力推动全国文明城市创建工作，城市管理、市民素质和市容市貌明显提升。群众性文体活动丰富多彩，市级送戏下乡800场，组织文化惠民活动1000余场；十三届全运会上我市运动员摘得5金2银，取得历史最佳成绩。

牢固树立“红线”意识和“底线”思维，全面开展“大排查、快整治、严执法、全资格”集中行动，安全生产形势保持稳定。平安德州持续推进，立体化社会治安防控体系建设深入实施，圆满完成十九大安保维稳任务，食品药品、信访、应急、市民热线等工作扎实开展。创新基层治理模式，全面推行城乡网格化管理，全市划分基本网格8358个，统筹落实综治维稳、公共安全、生态环保等基本任务，共调解矛盾纠纷7892件。国防动员、人民防空、民兵预备役和双拥工作成效明显，妇女、儿童、青少年、老年人和残疾人权益得到较好保障，民族宗教、对台事务、海关、口岸、检验检疫、档案、史志、防震减灾、慈善、红十字会等工作取得新进展。

（六）环保工作强力推进，生态建设成效显著

一年来，我们坚持不懈抓好污染治理，一批环境突出问题得到有效解决，整体环境质量再上一个新台阶。全年“蓝天白云、繁星闪烁”天数为240天，同比增加31.9%，SO2、PM10、PM2.5分别改善32.4%、16.3%、18.5%，NO2同比持平，“蓝繁”增加天数、PM10改善幅度、空气质量综合指数改善幅度实现了三个全省第一。组建了市生态环保委，齐抓共管的局面得到巩固。大力开展“散乱污”企业专项整治，采取31项措施精准控制扬尘，对4026家汽修企业开展排查整治。先后淘汰1192台10蒸吨以下燃煤锅炉，122台10蒸吨以上燃煤锅炉完成超低排放改造，超额完成省政府下达的节能减排任务，177家企业完成挥发性有机物治理。针对冬季采暖季节的污染问题，继续在农村大力度推广洁净型煤64万吨、环保炉具6.3万台，居全省前列，对6.7万户农村居民实施了“气代煤、电代煤”改造，对273家企业采取错峰生产调控，重污染天气应对能力显著增强。省环保厅在我市召开全省大气污染治理现场会，环保部在我市召开大气重污染成因与治理攻关“2+26”城市工作现场交流会，充分肯定了德州的经验做法。

大力度推进生态修复。统筹推进水污染防治、土壤污染治理、水生态保护，5条省控重点河流全面达到V类标准，集中式饮用水水源水质100%达标，完成了22条黑臭水体整治，完成禁养区161个规模养殖场和1145个养殖户的搬迁。封停各类地下水井957眼，压采水量3554万立方米，地下水生态环境有效改善。在全省率先开展“清河行动”，全面建立市县乡村四级河长体系，设立河长8449名，形成了“河长+警长+网格员”日常管护机制。实施“绿满德州生态美市”造林行动，植树2613万株；大运河生态林场完成一期建设，造林3100亩，绿意盎然，西陈沟省级生态湿地和东八里湿地顺利施工，水鸟点点，运河开发区生态修复已初见成效。一年来，环境生态不断改善，绿色发展深入人心，德州人民呼吸到了更干净的空气，喝到了更放心的水，看到了更多的蓝天白云，“德州蓝”正走向常态，日益成为我们城市发展的新优势！

（七）严格履行主体责任，政府自身建设不断加强

推进“两学一做”学习教育常态化制度化，全面贯彻落实党风廉政建设责任制各项要求，从严从实抓好市政府党组和政府系统党的建设。自觉接受人大工作监督、法律监督，主动加强与政协的民主协商，认真听取各民主党派、工商联、无党派人士和人民团体意见，共办理人大代表建议403件、政协委员提案605件。深入推进依法行政，完善科学民主依法决策制度。81%的乡镇（街道）建成公共法律服务站，3177个村（社区）配备了法律顾问，基层依法治理体系不断完善。

全面推进政务公开，加强行政监察和审计监督，强化绩效管理，开展行政问责。深入开展纠治“四风”联合行动，查处违反中央八项规定精神问题324起。坚定不移推进党风廉政建设和反腐败斗争，严肃惩处各种违纪违法行为，全市纪检监察机关共立查案件2670件，党纪政纪处分2500人，反腐倡廉取得新成效。

各位代表，过去的一年忙碌而充实，我们经受了很多考验，但始终保持定力，始终奋发图强，逢山开路，遇水架桥，工作局面来之不易。这是全市上下全面贯彻落实党中央、国务院和省委、省政府决策部署的结果，是市委统揽全局、坚强领导的结果，是市人大、市政协监督支持的结果，是全市各级干部特别是广大基层干部一年来辛勤工作、无私奉献的结果，是全市人民同心同德、共同奋斗的结果！在此，我代表市政府，向全市人民，向各位人大代表、政协委员、离退休老同志，向各民主党派、工商联、无党派人士和各人民团体，向驻德部队官兵，向中央、省驻德单位、各级新闻媒体，向所有关心支持德州发展的海内外朋友，致以崇高的敬意和衷心的感谢！

我们必须清醒认识到，德州正处在经济社会的转型期，人民群众日益增长的美好生活需要和不平衡不充分的发展之间的矛盾仍然十分突出。发展的质量还不够高，产业结构偏重，创新能力不强，实体经济发展还面临诸多困难；发展仍不够协调，城乡基础设施、公共服务、生态环保等方面还有较大差距；民生领域还有不少短板，群众收入水平还不高，不少群众的生活还比较困难，社会保障还需进一步提高；政府的决策水平、公共服务能力和工作效率均有待加强，“四风”及消极腐败现象依然不同程度存在等等。对上述问题，我们将本着对党和人民高度负责的态度，在今后的工作中认真推动解决。

二、深入学习贯彻党的十九大精神，全面开创新时代德州发展新局面

党的十九大是中国特色社会主义进入新时代的关键时期召开的一次具有里程碑意义的大会，把党的十九大精神和习近平新时代中国特色社会主义思想真正学懂弄通做实，贯彻到各方面、各领域工作中去，是我们当前和今后最核心的政治任务，是德州昂首走进新时代、谱写新篇章的根本保证。

（一）深刻把握习近平新时代中国特色社会主义思想的指导地位。党的十九大将习近平新时代中国特色社会主义思想确立为党长期坚持的指导思想，为中华民族的伟大复兴提供了坚强的政治保障和行动指南，是党心民心所向。我们要牢固树立“四个意识”，坚决维护习近平同志的核心地位和领袖权威，坚决同以习近平同志为核心的党中央保持高度一致。我们开展各项工作，都要自觉以习近平新时代中国特色社会主义思想为指导，不断深化对党的路线方针政策的理解，把十九大的各项要求转化为具体行动，不折不扣地在德州落地生根，坚定不移抓好贯彻落实。

（二）深刻把握新时代的历史方位。中国特色社会主义进入新时代，这是党中央作出的重大判断，是我国发展新的历史方位。新时代要有新气象，更要有新作为。我们要深刻领会新时代的丰富内涵，深刻把握社会主要矛盾的新变化，统筹谋划好德州未来的经济发展和社会建设，突出解决好发展不平衡不充分的问题，尽快缩短城乡之间、工农业之间、县域之间的差距。坚定不移走生产发展、生活富裕、生态良好的可持续发展道路，努力提高发展质量，增强城市综合实力，提高人民生活水平，在全面建成小康社会的征程中交出“德州答卷”。

（三）深刻把握新发展理念的要求。我国经济已由高速增长阶段转向高质量发展阶段，能否顺利完成产业结构升级关乎德州长远发展。贯彻落实“创新、协调、绿色、开放、共享”的新发展理念，建设现代化经济体系，是德州转变发展方式、优化经济结构、转换增长动力的根本路径。我们必须抓住战略机遇期，摒弃落后发展思维，着力推动经济发展质量变革、效率变革、动力变革，在改造传统产业、发展新兴产业、推动创新驱动上实现新突破，不断增强发展的协调性和可持续性。始终坚持“以开放引领德州发展”，持续扩大对内对外双向开放水平，提高城市整体开放意识，加快构建开放型经济新体制，努力营造公平的市场环境和法治环境，不断增强德州经济的创新力和竞争力。

（四）深刻把握以人民为中心的发展思想。全心全意为人民服务，是我们党的根本宗旨。我们发展的目的就是让老百姓过上更好的日子，我们必须顺应人民群众对美好生活的向往，把增进人民福祉、促进人的全面发展作为一切工作的出发点和落脚点，多谋民生之利、多解民生之忧，始终同全市人民想在一起、干在一起。民生保障始终是政府的中心工作，要坚持群众路线，多听群众意见，尊重群众意愿，努力实现“幼有所育、学有所教、劳有所得、病有所医、老有所养、住有所居、弱有所扶”，切实解决好与群众生活息息相关的问题，关心好群众身边的小事，不断提高公共服务质量，增加群众的幸福感和获得感。要维护好城市公共安全，维护社会和谐稳定，促进公平正义，畅通意见表达渠道，全力维护合法权益，给群众更多的安全感。

（五）深刻把握全面从严治党新要求。“全面从严治党永远在路上”，充分表明了我们党清醒认识形势、勇于直面问题的鲜明态度，进行自我革命、永葆生机活力的坚定自信，坚持不懈抓正风肃纪、反对腐败没有休止符的坚强决心。德州未来的发展根本在于坚持党的领导，坚持全面从严治党。我们要自觉在市委的正确领导下，不断提高政治觉悟，全面落实从严治党各项要求，把“严”字贯穿始终，狠抓政治建设、思想建设、组织建设、作风建设、纪律建设，让权力在阳光下运行，树立为民务实清廉的好作风，坚决治理“四风”问题，严惩贪污腐败行为，为推动各项事业发展提供政治保障和作风保障。

三、2018年重点工作

今年是贯彻落实党的十九大精神的开局之年，是改革开放40周年，是决胜全面建成小康社会、实施“十三五”规划承上启下的关键一年，做好今年工作具有重要意义。

2018年政府工作的总体思路是：深入学习贯彻党的十九大精神，以习近平新时代中国特色社会主义思想为指导，积极践行新发展理念，坚持稳中求进工作总基调，坚持高质量发展总要求，深入推进供给侧结构性改革和新旧动能转换重大工程，统筹推进稳增长、促改革、调结构、惠民生、防风险各项工作，深入实施协同发展战略，努力建设协同发展示范区，推动经济社会发展再上新水平。

主要经济预期目标为：地区生产总值增长7%左右，固定资产投资增长8%左右，一般公共预算收入增长6%左右，社会消费品零售总额增长9.5%左右，进出口稳定增长。城镇登记失业率控制在3.5%以内。居民消费价格涨幅控制在3%以内。城镇和农村居民人均可支配收入分别增长8%左右和8.5%左右。

重点做好六个方面工作：

（一）转变经济发展方式，着力推动高质量新发展

继续调整产业结构，大力破除无效供给。要树立鲜明的价值导向，统筹运用政策手段、市场规则和倒逼机制，坚决淘汰落后产能、改造低效产能、化解过剩产能。方向对了，不怕路远。要保持定力，不为一时的困难所干扰，对不符合要求的产业坚决不予审批，对落后的产能坚决不予输血，采取各种措施加快市场出清。要落实“三去一降一补”要求，出台的产业政策，鼓励什么、限制什么、禁止什么要清清楚楚，决不能含糊。要合理摆布产业资源，中心城区要列出产业退出清单，不符合城市生活要求的产业要坚决退出，铁路货场和物流园区等行业要逐步退出，全市14家在人口密集区的化工企业要全部启动搬迁。要以省级开发区为重点，开展闲置土地专项整治，采取各种方式盘活“休克”企业占用的土地资源，使开发区更好发挥经济主引擎的作用。要继续限制高耗能产业的发展，力争全年煤炭消费量控制在1880万吨以内，完成万元GDP能耗再下降3.66%的省控目标。

实施“传统产业提升”计划，提升产业发展质量。坚持质量第一、效益优先，推动传统产业现代化、智能化、品牌化，不断做大做强。要大力引导企业树立现代经营管理理念，建立现代企业制度，摆脱传统路径依赖，树立市场思维、法治观念和规则意识，走可持续发展之路，这是我市传统产业升级的根本和关键。要深入实施“三航”计划，进一步划清政府“有形之手”的权力边界，以公共服务的方式为骨干企业提供低成本、契约化的金融服务，形成规范健康的政企互动关系。要推进企业技术改造，全年实施150个工业重点技改项目，鼓励企业采用新技术、新工艺、新装备，力争用三年时间帮助主导产业规上企业进行一轮高水平技术改造。积极推进工业智能化，以互联网思维改造传统产业，建立中小企业云服务平台，让大数据为更多企业提供服务。要着力培育“专精特新”企业和行业“隐形冠军”，支持在细分市场深耕细作的“小巨人”企业，打造充满活力的中小企业群。全方位树立质量第一意识，加大品牌创建力度，推进“德州标准”建设，力争各县（市、区）马德里商标国际注册实现全覆盖，叫响“德州品牌”。

实施“新兴产业倍增”计划，大力培育新动能。坚持以“四新”促“四化”，全年力争战略性新兴产业增加值占生产总值比重提高1个百分点。加快建设东部城区德州创新谷、高新区（禹城）中央创新区、齐河中关村海淀高新园区，形成我市三大创新集群区，集中承载科技型、创新型产业，积聚德州未来产业发展新优势。新能源汽车、节能环保、生物技术、新材料、电子信息等战略性新兴产业都要加快发展，氢燃料电池等前沿性技术要加快转化应用。支持高新技术企业成长，力争全年新认定省级以上高新技术企业50家，高新技术产业产值增长18%，高新技术产业产值累计占规模以上工业总产值比重增加1.3个百分点，规模以上工业企业研发投入力争达到60亿元。新建省级以上企业技术中心、工程技术研究中心等科技创新平台30家以上，新增重大科技成果转化项目100个左右，与省科技厅共建山东省技术转移转化中心和省技术经理人培训学院。突出人才支撑作用。加快鲁北人才改革试验区建设，优化政策措施，务实引入各类人才，持续增加城市人才总量。发挥区位优势，鼓励企事业单位引进周边大城市“周末工程师”来德州创新创业，不拘一格吸引人才。

（二）推进城乡建设，着力构建协调发展新格局

全面启动东部城区建设。高水平开发建设东部城区，是市委、市政府作出的重大决策，是着眼城市长远发展的战略举措，是培育发展新优势、拓展城市新空间的现实需要，是德州的百年大计。我们要坚持规划引领，以“宜居宜业的现代化新城”为目标，以世界眼光、国际标准，前瞻性规划新区功能。要迅速启动建设，以德州创新谷为核心的科技组团，以德州国际会展中心为核心的商务组团，以东部医疗中心、养老城、高级师范学校为核心的公共服务组团，以杨庄河生态公园、太阳能德州小镇、科技公园为核心的生态组团，以京津冀产业合作区为核心的产业发展组团，以百亩级智慧大棚群为核心的现代农业发展组团等六个组团要率先启动，一批基础性、支撑性项目要率先建设。要以高度的历史责任感做好东部城区的开发建设工作，打造精品工程，不留历史遗憾，一张蓝图干到底，尽快形成引领全市发展的新增长极。

进一步提高城市规划建设水平。启动新一轮城市总体规划修编，牢固树立“规划即法”意识，今年实现主城区控规全覆盖。以“生态修复、城市修补”为目标，重点实施5大类80个中心城区公共类城建项目，年度投资135亿元。集中实施雨污分流改造工程、棚户区、老旧小区改造和公共租赁住房建设，大规模开展城市绿化美化亮化，不断提升城市环境质量。持续优化城乡路网结构，增加主城区对外通道，加快构建内畅外联互通的交通体系。开工建设德上高速京台高速至国道105段、京台高速德州至齐河段改扩建，启动幸福大道南延工程；实施乐陵、平原、庆云等3个绕城国省道改造；建设高铁站东站房，完成城市轻轨线规划工作。中心城区再增加新能源公交车100辆，建成500处乡村客运站点，实现具备条件的建制村全部通客车。大力推进“四好农村路”建设，既要把农村公路建设好，更要管护好、运营好。完善水网工程体系，开展潘庄、李家岸灌区改造，推进引位（河）入德工程，实施市域水系连通工程。全面提升城乡污水处理设施，保障316个乡镇驻地和农村新型社区污水处理项目正常运行。大力加强电力、燃气、供热等能源体系建设，推动农村电网改造升级；提升县城集中供暖能力，有序推进农村清洁能源供暖改造。

（三）做好“三农”工作，着力培育农业发展新动能

大力实施乡村振兴战略。乡村振兴战略是新时代做好“三农”工作的总抓手，我们要认真学习落实中央的工作部署，把农业农村优先发展的要求落到实处，按照“产业兴旺、生态宜居、乡风文明、治理有效、生活富裕”的总要求，统筹把握城乡关系，集中更多资源投入到农村，大力推进美丽乡村建设，完善农村公共基础设施，改善农村人居环境，提高我市农业竞争力和农民收入水平，完善农村社会保障体系，加强扶贫工作，创新和加强乡村治理。特别要探索一条传统粮食产区发展现代农业的有效路径。继续深化供销合作社综合改革，加快为农服务中心建设，力争2018年实现涉农乡镇全覆盖，扎实推进党支部领办创办土地股份合作社。强化科技对农业的支撑作用，大力发展新业态，培育新型职业农民，激发农业发展活力，培养造就一支懂农业、爱农村、爱农民的“三农”工作队伍，推动我市农业全面升级、农村全面进步、农民全面发展。

加快农业供给侧结构性改革。坚持质量兴农、绿色兴农，把农业结构调好调顺调优，加快农业由增产导向转向提质导向。全面推进“放心农场”建设，促进小农户和现代农业发展有机衔接，推进“互联网+现代农业”，全面提升农产品质量安全水平。稳定粮食生产，实施“优质粮食工程”，加快规模化、标准化现代农业园区建设，以30个百亩级智慧农业大棚群为核心的现代农业示范区、武城昌盛日电大型农光互补项目、广东温氏集团养猪项目、美国雅培高端牧场等重点农业项目要加快建设，农业国际合作要务实推进。着力打造一批高标准千亩蔬菜示范片、万亩示范区和专业特色镇，力争新增专业蔬菜园区15个以上，新增设施蔬菜5万亩，蔬菜产量突破1300万吨。大力发展规模高效养殖，新增和提升标准化规模养殖场（区）200处以上。积极发展农业“新六产”，大力扶持和引进农业龙头企业，提高农业附加值，推动农业全环节提升、全链条增值、全产业融合。

（四）继续深化改革，着力拓展开放新空间

扎实推进各项改革落地。继续深化“放管服”，推动“市权县用”，构建扁平化审批服务机制，提高行政效能。优化整合办事流程，实现“全链条”办理、“一站式”办结，让群众办事更快捷、更便利。加强市场主体监管，建立全市统一的监管平台，出台共享单车等新业态监管办法。继续推进工商登记全程电子化和电子营业执照等商事制度改革。创新规范各级开发区管理体制和运行机制，有序推进环保、盐业、综合行政执法体制改革。支持禹城开展国家土地制度三项改革，深化事业单位分类改革，推进政事分开、事企分开、管办分离。理顺市县医改管理体制，抓好公立医院法人治理结构建设，建立现代医院管理制度。落实最严格水资源管理制度，积极推进农业水价综合改革，促进集约节约使用水资源。做好国有林场改革的政策衔接和落实。

加快构建全面开放新格局。坚持对内对外双向开放，牢牢把握京津冀协同发展、雄安新区建设等重大机遇，健全完善专业化对接机制，全面对接京津，主动融入省会，南北双向借力，提高协同发展的质量。要以开放的主动赢得发展的主动，持续拓展东盟市场，机制化开展“德州百企下南洋”活动，引导有条件的企业走出去，举办好中国—东盟中小企业合作高峰会等重要商务活动，努力使德州成为中国北方对接东盟的重要节点城市。全力办好第二届中国国际太阳能十项全能竞赛。拓展对外贸易，扶持一批外贸支柱企业，加快发展海外仓、跨境电商等新业态新模式，促进全市对外贸易实现跨越增长。拓宽企业家开放视野，年内组织全市制造业50强和创新型企业30强主要负责人赴美国、德国对标学习，努力培育具有国际合作能力的企业家队伍。积极拓宽与欧美、日韩以及港澳台等区域的合作渠道，加强与国家、省驻外机构对接，健全完善与世界500强企业合作行动方案，对重点跨国公司开展精准对接，提升利用外资水平。注重发挥国际友好城市和海外侨社组织的桥梁纽带作用，不断拓展对外开放的广度和深度，开创高水平对外开放新局面。

（五）注重改善民生，着力提高保障新水平

坚决打赢脱贫攻坚战。牢固树立以人民为中心的发展思想，持续巩固脱贫成效。严格落实“两不愁、三保障”的要求，在确保高质量完成年度脱贫目标的同时，建立防止返贫长效机制，加大大病保险、困难救助等帮扶力度，决不让一个贫困群众掉队。继续强化产业扶贫，巩固老项目、发展新项目、培育大项目，以产业项目带动村集体经济发展和群众持续增收。严格扶贫资金监管，扶贫资金是贫困群众的“救命钱”，一分一厘都不能乱花。完善督导考核和激励约束机制，压实各级责任，防止“挂帅不出征、挂名不扶贫、包村不入户”的问题，防止弄虚作假和形式主义，确保脱贫成效经得起检验。注重扶贫同扶志、扶智相结合，既让贫困群众在经济上脱贫，更要在面貌上改善。

着力办好人民满意的教育。扩大优质和多样的教育供给，着力办好“公平而有质量”的教育，在巩固义务教育“基本均衡”的基础上，加快向“优质均衡”迈进，提高群众对教育的满意度。“全面改薄”工作要继续巩固提升，“大班额”问题要展开攻坚，今年要新建、续建学校33所，新增学位5万个。要加强师德教育，优化教师结构，不断改善基层办学条件。继续实施学前教育三年行动计划，加强普惠性幼儿园建设，切实解决“入园难”、“入园贵”、婴幼儿照护和儿童早期教育等问题。增加我市普通高中学校的数量和学位，均衡优质普通高中资源，形成良性竞争态势。重视发展职业教育，抓好省级规范化中等职业学校建设。鼓励与京津冀高水平学校的合作，努力让德州孩子在家门口能上更好的学校，接受更好的教育。

大力发展卫生健康事业。加大投入，补齐优质医疗资源不足的短板，促进卫生计生事业更充分发展。加快东部医疗中心等市级项目和8个县级重点医疗卫生项目建设，力争三年内增加床位1万张。提升市县两级医疗服务能力，实施“中青年医学专家培养计划”“基层医务人员培训计划”，抓好医院院长和医技人员队伍建设。重点实施医疗、医保、医药“三医联动”，推进医疗机构稳定健康发展。加大医联体、医共体建设力度，加快推动分级诊疗。注重提高精神疾病的收治能力，加快市县两级专业医院建设，健全传染病防治和卫生应急工作体系。全面落实计划生育家庭发展政策，促进人口均衡发展。积极发展医养融合型的养老机构，新增养老床位4000张。广泛开展全民健身活动，增加体育健身设施，更好满足市民体育健身需求。

努力提高社会保障能力。继续实施技能培训计划，支持“大众创业、万众创新”，落实创业补贴、创业担保贷款等政策，全年新增城镇就业5.8万人。全面实施全民参保计划，实现城乡居民医疗保险基金市级统筹。调整机关事业单位工作人员基本工资标准。调整最低工资标准，提高职工工资水平，增加居民财产性收入。加快住房保障建设，中心城区老旧小区改造三年攻坚任务要全部完成，6万套棚户区改造要全面开工，农村危房改造做到应改尽改。坚持“房子是用来住的、不是用来炒的”定位，保持房地产市场健康平稳运行，坚决防止房价过快增长，坚决打击房地产领域的欺诈行为，坚决维护广大老百姓的切身利益。做好城乡低保、特困人员保障工作，构建制度化、规范化、程序化的幸福救助体系。

持续改善生态环境质量。牢固树立“绿水青山就是金山银山”的理念，巩固好来之不易的生态治理局面，以钉钉子的精神将生态文明建设推向深入。要继续打好大气污染防治攻坚战，严格控制燃煤总量，已取缔的小锅炉要防止反弹，大型工业锅炉要加强排放监管；加大扬尘治理力度，做好城市保洁，加强建筑工地等管理，精细化落实属地责任；机动车排放要继续严管，合理规划，重点解决重型车辆穿城问题；深入推进各行业挥发性有机物治理和提标改造，全面推广餐饮行业高效油烟净化设施建设；加快实施大运河生态林场二期等11项市重点生态修复工程，持续提升城市绿化水平。要深化企业主体责任，将排污情况向社会公开，接受监督。要完成我市农村清洁能源替代规划，因地制宜，实事求是确定冬季清洁供暖方式，用五年左右的时间完成农村清洁能源替代，今年继续大力度推动“气电代煤”和清洁煤的推广工作。要做好水污染防治工作，全面落实河长制要求，继续推进“清河行动”，对饮用水水源地进行集中整治，启动中心城区“三河”水生态循环工程，确保国控、省控断面水质稳定达标。实施城乡污水处理设施建设、雨污分流改造、黑臭水体治理、地下水开采控制等重点工作，推进我市主要河流水环境质量提标升级。做好土地污染治理工作，加强农业面源污染防治，提高危险废物规范化管理水平和处置利用能力。不断提高环境治理和监管水平，强化环保人才、科技、装备支撑，完善监测网络，加大部门联动、行刑衔接和综合执法工作力度，严厉打击各类环境违法行为，营造全社会参与环境共治的氛围。

推动文化繁荣发展。以“我文明德州文明”为主题，广泛开展精神文明创建活动。积极构建现代公共文化服务体系，加快推进市级文化场馆建设，鼓励有条件的县（市、区）建设综合文化中心，实现基层综合性文化服务中心村级全覆盖。全面实施文化惠民工程，继续组织公益演出下基层，大力推动全民阅读，组织开展丰富多彩的群众文化活动。切实加强文化遗产保护传承，启动“大运河保护建设工程”，使大运河德州段“动起来、活起来、美起来、富起来”，成为值得骄傲的城市名片。加强文物保护基层基础工作，推进新闻出版、广播影视改革发展，加强文化市场和网络空间监管。编制全市文化产业发展规划，培育一批文化产业新型项目，打造特色文化品牌。

加强和创新社会治理。坚决打好防范化解重大风险攻坚战。牢固树立安全发展理念，强化安全生产责任落实，深化重点领域安全工作，坚决遏制较大及以上事故发生。大力实施食品安全战略，严防严管严控食品药品安全风险。着力推进金融风险防控，完善金融风险监测预警机制，抓好重点领域、重点行业的风险防范化解，坚决打击恶意逃废债务行为，严厉打击非法集资和互联网金融犯罪，规范政府债务行为，坚决守住不发生系统性金融风险的底线。全力维护社会稳定。始终保持对各类违法犯罪的严打态势，实施“雪亮”工程，完善联动协作系统，加强社会治安防控体系建设，推动城乡网格化管理与社区警务工作有效融合，提高基层社会治理社会化、法治化、智能化、专业化水平，打造共治共建共享的社会治理格局。大力推进公共法律服务体系建设，认真做好信访工作，及时回应群众合理诉求，妥善化解各类矛盾，努力建设平安德州。

（六）加强政府自身建设，着力提升队伍新担当

坚持把党的建设摆在首位。牢固树立“四个意识”，强化理想信念，始终坚持正确的政治方向，把抓好党建作为最大政绩。认真贯彻落实上级和市委的各项决策，扎实开展“不忘初心、牢记使命”主题教育，引导党员干部永葆政治本色。要牢记根本宗旨，自觉把群众路线贯穿政府工作各领域全过程，全心全意为群众办好事、做实事。要严格主体责任，加强作风建设，深入落实中央八项规定精神，驰而不息纠治“四风”，大力整治形式主义、官僚主义，强化监督执纪问责，坚决惩治各种形式的腐败行为，树立风清气正的政治生态环境。

坚持依法全面履职。构建决策科学、执行坚决、监督有力的权力运行机制。依法接受人大及其常委会的法律监督和工作监督，认真执行有关决议决定，主动报告政府工作。加强同人民政协的民主协商，接受民主监督，认真听取各民主党派、工商联、无党派人士和人民团体的意见建议。全力支持国家监察体制改革试点工作，自觉接受监察委员会的监督。加强政府内部监督，推进审计监督全覆盖。深入推进政务决策、执行、管理、服务和结果全过程公开。加强法律学习，完善法律顾问制度，自觉运用法治思维和法治方式加快改革、推动发展、化解矛盾，把政府工作全面纳入法治轨道。

坚持不断加强队伍建设。着力增强“八种本领”，把提高政府系统专业素养摆在更加突出位置，加强知识更新和培训，打造高素质专业化的工作队伍，提高领导经济工作的本领和解决实际问题的能力。幸福都是奋斗出来的，德州的发展更是广大干部群众艰苦创业干出来的。要大力弘扬干事创业的作风，坚决反对庸政懒政、求稳怕乱、明哲保身、不作为乱作为。要健全正向激励、容错纠错机制，鼓励干部尚实干、勇作为、敢担当，旗帜鲜明地为踏实做事、不谋私利的干部撑腰鼓劲。

各位代表，新时代有新召唤，要迈出新步伐，让我们更加紧密地团结在以习近平同志为核心的党中央周围，以习近平新时代中国特色社会主义思想为指引，不忘初心，牢记使命，从新的历史起点出发，全力推进协同发展示范区建设，在新时代宏阔画卷上绘就德州更加辉煌的明天，大踏步迈向更加美好的未来！
